# Comment on “Gas Bubbles From Biodegradable Magnesium Implants Convey Mechanical Cues and Promote Immune Cell Stimulation”

**DOI:** 10.1002/advs.202518129

**Published:** 2025-11-04

**Authors:** Lian Cui, Jiangluyi Cai, Hengli Lu, Xincheng Huang, Yuling Shi, Chunyuan Guo

**Affiliations:** ^1^ Department of Dermatology, Shanghai Skin Disease Hospital Tongji University School of Medicine Shanghai 200443; ^2^ Institute of Psoriasis Tongji University School of Medicine Shanghai 200443 China; ^3^ Department of Orthopedic Oncology, Shanghai General Hospital Shanghai Jiao Tong University School of Medicine Shanghai 200025 China

Recently, a study by Ben Amara et al. in *Advanced Science* has provided a novel perspective on the biological significance of gas bubbles generated during magnesium implant degradation.^[^ ^1^ ^]^ This study investigates how gas bubbles formed during magnesium degradation influence surrounding tissues in a site‐specific manner, providing important evidence to address the previously debated effects of degradation‐induced bubbles on tissue responses.^[^ ^2^ ^]^ The authors demonstrate that bubbles act as bioactive physical structures, orchestrating spatiotemporally defined inflammatory and regenerative responses. By integrating advanced imaging, immunohistochemistry, and spatial transcriptomics, they show that bubble formation is not inert but rather a dynamic component of host–biomaterial interactions, with profound implications for the design and clinical translation of biodegradable implants. Although bubble accumulation around magnesium implants has been observed since their earliest medical applications over a century ago, its biological significance has remained unclear. The prevailing belief has been that bubbles hinder healing and impair implant performance; however, emerging evidence—particularly regarding the potential modulatory effects of hydrogen (H_2_)—now challenges this view, suggesting that bubbles may exert multifaceted, context‐dependent influences on tissue repair.

One of the most striking findings of this work is the demonstration that gas bubbles produced by magnesium implants affect both inflammation and healing. Bubble formation peaked around day 3 and resolved by day 28, with the densest accumulation on the deep side of implants. Strikingly, immune cells clustered most densely within 20 µm of the bubble rim—often exceeding those at the implant surface itself. The peribubble niche was enriched with macrophages (cluster of differentiation 68(CD68^+^)) displaying both pro‐inflammatory (inducible nitric oxide synthase (iNOS^+^)) and pro‐repair (mannose receptor C‐type 1(MRC1^+^)) phenotypes, alongside elevated Piezo Type Mechanosensitive Ion Channel Component 1 (PIEZO1) expression, highlighting a strong mechanosensitive response. Spatial transcriptomics further revealed upregulation of inflammatory pathways, such as neutrophil degranulation and innate immune signaling, together with cytoskeletal regulators (Secreted Phosphoprotein 1 (SPP1), Myosin IG (MYO1G), Actin Related Protein 2/3 Complex Subunit 2 (ARPC2), Dedicator of Cytokinesis 8 (DOCK8)), whereas extracellular matrix assembly programs were suppressed at the bubble interface. Importantly, these highly localized signatures were undetectable by bulk assays, underscoring the unique biology of the peribubble microenvironment. Importantly, *SPP1*—a key gene elevated in the bubble niche—has been widely implicated in coordinating macrophage function during tissue repair, further highlighting the similarity between bubble‐induced responses and early wound healing. Immune activation intensified as bubbles formed farther from the implant, coinciding with reduced engagement of regenerative extracellular matrix (ECM) programs—creating a spatial dichotomy between immune‐dominant distant niches and matrix‐dominant implant interfaces. Functionally, bubbles behave as persistent micro‐cavities that sustain immune activation until resorption, consistent with their eventual disappearance within four weeks. Collectively, these findings suggest that bubbles may act as “mechanical wounds”, disrupting local tissue architecture, recruiting and activating immune cells, which closely mimic the features of wound healing.

Although the physical properties of gas bubbles are central to their biological impact, the potential contributions of their chemical composition also warrant careful consideration. Magnesium corrosion predominantly produces H_2_, yet the actual in vivo bubble composition is likely mixed.^[^ ^2^ ^]^ Traditionally, H_2_ has been regarded as biologically inert; however, growing evidence reveals its ability to modulate redox homeostasis and suppress inflammation, suggesting that gas bubbles may exert both mechanical and chemical influences.^[^ ^3^ ^]^ Recent studies demonstrate that molecular H_2_ enhances epidermal stem cell proliferation and extracellular matrix deposition, thereby accelerating cutaneous wound healing.^[^ ^4^ ^]^ Moreover, H_2_ facilitates vascular regeneration and mitigates pathological neovascularization and neuroglial dysfunction in retinal injury models, underscoring its broad potential in tissue repair.^[^ ^5^ ^]^ Emerging data further indicate that H_2_ released during magnesium degradation serves as a bioactive mediator that synergizes with local physicochemical cues to promote regeneration.^[^ ^6^ ^]^ In addition, other gaseous byproducts, such as carbon monoxide (CO) and carbon dioxide (CO_2_), may also be generated during magnesium implant degradation in vivo,^[^ ^2^ ^]^ although some studies have detected only H_2_ and CO_2_.^[^ ^7^ ^]^ At high concentrations, both CO and CO_2_ can exert cytotoxic or pro‐inflammatory effects, with CO potentially disrupting oxygen transport and CO_2_ lowering local pH and perturbing cellular function. Conversely, at physiological or controlled levels, CO can act as an endogenous signaling molecule to modulate inflammation and promote tissue repair,^[^ ^8^, ^9^, ^10^ ^]^ while CO_2_ may enhance local blood flow and oxygen delivery, thereby supporting regeneration.^[^ ^11^, ^12^, ^13^ ^]^ Nonetheless, the precise gas composition and its potential biological implications remain insufficiently characterized, highlighting the need for further systematic investigations using advanced in situ analytical techniques. Beyond gaseous products, corrosion processes also alter the local ionic environment and pH, indirectly shaping immune activation. Collectively, while mechanical stress appears to be the dominant driver of bubble‐induced responses, localized chemical fluxes likely act in synergy with physical cues to reinforce immune recruitment and modulate the balance between inflammation and regeneration. Moreover, trace degradation products may diffuse locally via gas bubbles, potentially influencing adjacent stromal and immune cells. Advanced in situ techniques, such as Raman spectroscopy, could help determine whether bubbles carry specific chemical signatures, thereby further supporting the notion that bubble‐induced biological responses arise from an interplay of mechanical, chemical, and spatial cues, rather than purely physical effects.^[^ ^14^, ^15^, ^16^ ^]^

Beyond the bubbles themselves, these findings raise broader questions: do soft or dynamic physical cues elicit immune responses differently compared to rigid intrusions? Rigid challenges—such as rigid foreign bodies, non‐degradable implants (e.g., titanium or polymers) or surgical microinjuries—are well known to induce persistent inflammation, often culminating in frustrated phagocytosis and fibrous encapsulation at rigid surfaces.^[^ ^17^ ^]^ In contrast, bubbles—although not classical “soft matter” in the strict sense—share key characteristics with soft matter systems: they are governed by interfacial tension rather than bulk mechanics, highly deformable under external forces, and, when forming foams, display complex multiscale dynamics akin to colloids and emulsions.^[^ ^18^ ^]^ Being soft, mobile, and ultimately resorbed, bubbles act as transient mechanical cues more likely to trigger short‐term regenerative pathways rather than chronic inflammation.^[^ ^19^ ^]^ Supporting this notion, immune cells such as macrophages cultured on softer hydrogel substrates show a differential inflammatory gene expression profile compared with those on stiffer matrices, suggesting that “soft” mechanical environments, such as those created by gas bubbles, can modulate immune behavior.^[^ ^20^ ^]^ Exogenous air bubbles exemplify this principle: they elicit mechanosensitive recruitment through interfacial tension and hypoxia gradients, yet without continuous chemical flux, they resolve rapidly, leaving only weak immune imprints. Notably, magnesium‐derived bubbles, in contrast, are unique in combining sustained mechanical cues with corrosion‐driven chemistry, producing persistent and distance‐dependent immune activation. Taken together, these comparisons highlight how the immune system discriminates between rigid, soft, transient, and chemically active physical intrusions, translating them into qualitatively distinct inflammatory and regenerative outcomes.

Mechanistically, this study highlights the pivotal role of PIEZO1 in mediating bubble‐induced immune modulation. PIEZO1, a mechanosensitive ion channel, has been experimentally shown to regulate macrophage polarization, T cell differentiation, and broader immune responses.^[^ ^21^, ^22^, ^23^, ^24^, ^25^, ^26^ ^]^ Loss of PIEZO1 in macrophages attenuates inflammatory activation and enhances wound healing, while toll‐like receptor 4 (TLR4) signaling through PIEZO1 has been shown to promote phagocytosis.^[^ ^22^, ^25^ ^]^ Similarly, in T cells, PIEZO1 activation drives calcium influx and integrin clustering, thereby facilitating directed migration.^[^ ^26^ ^]^ Notably, mechanical stress generated by bubbles may also activate PIEZO1 in stromal cells such as fibroblasts, enhancing chemokine secretion and establishing gradients that orchestrate immune cell recruitment. This stromal–immune crosstalk extends the regulatory influence of bubble‐mediated effects beyond immune cells alone. Future studies employing PIEZO1 inhibitors or conditional knockout models are warranted to define quantitative thresholds and fully delineate this mechanotransduction pathway.

Another notable contribution of this study lies in its methodological innovation. By applying spatially resolved transcriptomics, the authors were able to map both cellular composition and gene expression within the peribubble microenvironment, thereby providing a high‐resolution view of how immune cells respond to mechanical and biochemical cues generated by gas bubbles. This approach addresses a critical gap in biomaterials research by enabling detailed characterization of immune activation and mechanotransduction processes, moving beyond the limitations of imaging‐based observations alone. Furthermore, this methodology offers broad applicability for probing how degradation products from other bubble‐generating or biodegradable implants influence surrounding tissues and local cellular responses, providing a versatile platform for advancing our understanding of host–biomaterial interactions beyond magnesium‐based systems.

Bubble‐induced immune activation is highly context‐dependent and may not always be beneficial. In the early days after surgery, bubbles can compress surrounding tissues, recruit immune cells, and sustain local inflammation. In most cases, these cavities resolve within weeks, allowing healing to progress normally. However, if the bubbles persist or accumulate excessively, they may trigger complications such as chronic inflammation and tissue damage. In particular, excessive macrophage activation and cytokine release could shift the balance away from repair toward deleterious inflammation. Defining the thresholds of bubble formation—potentially in terms of bubble size, volume, and density per implant area—and elucidating the spatiotemporal dynamics of bubble‐associated immune activation will therefore be critical for optimizing host responses. Establishing such quantitative parameters could also facilitate the development of clinically relevant monitoring strategies and guide future implant design. Furthermore, while this study focuses primarily on macrophages, other immune populations—including neutrophils, dendritic cells, and adaptive lymphocytes—should be considered to achieve a more comprehensive understanding of host–bubble interactions. Validation in large animal models and ultimately human studies is also essential, as implant degradation kinetics and immune landscapes are likely more complex than in small animal models.

Future investigations should also leverage advanced biophysical tools to dissect the mechanistic underpinnings of bubble‐induced signaling. Techniques such as atomic force microscopy (AFM) and traction force microscopy (TFM) could be applied to systematically investigate how bubbles deform cell membranes and trigger downstream signaling events, including calcium flux and cytoskeletal remodeling. AFM has been widely employed to measure local membrane elasticity and reveal cellular responses to mechanical stimuli, while TFM enables quantitative analysis of cell–matrix traction forces, providing insight into how cells sense and respond to external mechanical cues. The integration of AFM with live‐cell calcium imaging has already demonstrated how mechanical perturbations evoke calcium signaling in neurons, underscoring its potential utility for studying immune and stromal cells in the peri‐bubble niche. Harnessing these approaches would offer a powerful strategy to dissect bubble‐induced mechanotransduction at the single‐cell level.^[^ ^27^, ^28^ ^]^

From a translational perspective, systematic studies correlating bubble size and frequency with immune activation will be essential to define safe and therapeutic windows for clinical applications. Material engineering strategies, such as alloy optimization or surface modification, may allow precise control of bubble formation, thereby achieving tunable immunomodulation. In addition, integrating computational modeling with experimental validation, particularly using advanced artificial intelligence (AI) ‐based predictive approaches, could help forecast in vivo bubble dynamics and host responses, ultimately providing a rational basis for personalized implant design and therapeutic intervention.

In conclusion, Ben Amara et al. present an elegant and forward‐thinking study that redefines the biological significance of biodegradable magnesium implants. By demonstrating that gas bubbles are biologically active participants rather than inert byproducts, this work uncovers a previously underappreciated interface linking biomaterials science, biomechanics, and immunology. Although challenges remain in delineating and balancing their beneficial and detrimental effects, the concept of harnessing degradation‐induced mechanical cues as therapeutic signals opens exciting new avenues in regenerative medicine. Magnesium implants, traditionally considered purely structural supports, may evolve into dynamic regulators capable of orchestrating immune modulation and tissue repair.

## Conflict of Interest

The authors declare no conflict of interest.

## Author Contributions

L.C. and J.C. authors contributed equally to this work.
